# Differential parameters between activity flare and acute infection in pediatric patients with systemic lupus erythematosus

**DOI:** 10.1038/s41598-020-76789-6

**Published:** 2020-11-16

**Authors:** Kai-Ling Luo, Yao-Hsu Yang, Yu-Tsan Lin, Ya-Chiao Hu, Hsin-Hui Yu, Li-Chieh Wang, Bor-Luen Chiang, Jyh-Hong Lee

**Affiliations:** 1 Department of Pediatrics, Cathay General Hospital, Taipei, 10630 Taiwan, ROC; 2 Department of Pediatrics, National Taiwan University Hospital and National Taiwan University College of Medicine, 8 Chung-Shan South Road, Taipei, 10002 Taiwan, ROC; 3 Graduate Institute of Clinical Medicine, National Taiwan University College of Medicine, Taipei, 10002 Taiwan, ROC

**Keywords:** Immunology, Biomarkers, Rheumatology

## Abstract

Systemic lupus erythematosus (SLE) patients are vulnerable to infections. We aim to explore the approach to differentiate active infection from disease activity in pediatric SLE patients. Fifty pediatric SLE patients presenting with 185 clinical visits were collected. The associations between both clinical and laboratory parameters and the outcome groups were analyzed using generalized estimating equations (GEEs). These 185 visits were divided into 4 outcome groups: infected-active (n = 102), infected-inactive (n = 11), noninfected-active (n = 59), and noninfected-inactive (n = 13) visits. Multivariate GEE (generalized estimating equation) analysis showed that SDI, SLEDAI-2K, neutrophil‐to‐lymphocyte ratio (NLR), hemoglobin, platelet, RDW-to-platelet ratio (RPR), and C3 are predictive of flare (combined calculated AUC of 0.8964 and with sensitivity of 82.2% and specificity of 90.9%). Multivariate GEE analysis showed that SDI, fever temperature, CRP, procalcitonin (PCT), lymphocyte percentage, NLR, hemoglobin, and renal score in SLEDAI-2k are predictive of infection (combined calculated AUC of 0.7886 and with sensitivity of 63.5% and specificity of 89.2%). We can simultaneously predict 4 different outcome with accuracy of 70.13% for infected-active group, 10% for infected-inactive group, 59.57% for noninfected-active group, and 84.62% for noninfected-inactive group, respectively. Combination of parameters from four different domains simultaneously, including inflammation (CRP, ESR, PCT), hematology (Lymphocyte percentage, NLR, PLR), complement (C3, C4), and clinical status (SLEDAI, SDI) is objective and effective to differentiate flares from infections in pediatric SLE patients.

## Introduction

Systemic lupus erythematosus (SLE) is an autoimmune disease caused by autoreactive B cells in combination with T cell dysregulation and cytokine abnormalities^1^. The presentation, disease course, and outcomes of SLE are unpredictable. Approximately 60–70% of patients exhibit relapsing–remitting and active disease patterns^2^. Pediatric lupus patients typically have a severe disease course. Additionally, in comparison to adults, a significantly higher percentage of children with SLE continue to have a status of high disease activity^3^. Several indices have been designed to assess disease activity. The most commonly used disease activity score is the Systemic Lupus Erythematosus Disease Activity Index (SLEDAI)^4^. SLE can also be complicated by chronic multiorgan damage. The Systemic Lupus International Collaborating Clinics/American College of Rheumatology Damage Index (SDI) is a reliable instrument for the assessment of the degree of disease-related damage in children with SLE^5^.

SLE patients are highly susceptible to infections due to the combined effects of immunosuppressive therapy and immune system abnormalities. In Taiwan, infections are among the leading causes of death in pediatric SLE^6^. Moreover, fever is a common symptom in pediatric SLE, and it is difficult to distinguish between an SLE flare and febrile infection^7^. Some infections may produce systemic manifestations mimicking SLE, either superimposed upon or triggering a flare^8^, making the diagnosis and therapeutic approach challenging. In one study, a delay in antimicrobial therapy of > 24 h reportedly increased the mortality of hospitalized SLE patients 12-fold; therefore, early identification and treatment of infections are essential^9^. The interaction between infection and SLE is complicated, as viral, bacterial, parasitic, and fungal pathogens can trigger SLE disease activity through molecular mimicry^10^. The establishment of a causative link between infection and autoimmunity has been studied in detail, confirming the role of infectious agents in the induction as well as the progression or exacerbation of SLE^11^. In general, clinicians have to make treatment decisions based on clinical judgment and laboratory parameters to distinguish between active disease and infection. Most such studies to date have been performed in adult populations, whereas data regarding pediatric SLE are lacking.

There have been a number of studies on predictive biological markers of SLE flares, including anti-double-stranded DNA antibodies (anti-dsDNA Ab), the complement system, anti-extractable nuclear antigen antibodies (anti-ENA Ab), cytokines, and chemokines^12,13^. In addition, conventional biomarkers (C-reactive protein [CRP], erythrocyte sedimentation rate (ESR), procalcitonin [PCT])^14–18^ and new markers have been developed for the prediction of infection in SLE patients^19^. Although several recent studies have focused on markers for differentiating between disease flare and infection in febrile SLE patients^20–22^, most physicians agree that no single biomarker has sufficient predictive value for both events^8,19^. The neutrophil-to-lymphocyte ratio (NLR) and platelet-to-lymphocyte ratio (PLR) are significantly higher in SLE patients than in healthy controls and correlate positively with the SLEDAI score^23^. However, the NLR might be a good additive marker for diagnosing infection in patients with SLE^24^. New scores, which include combinations of different biomarkers, may represent better solutions for differentiation^19^.

Overall, it is possible that the use of only one biomarker would not be sufficient to distinguish infection from disease activity. We aimed to identify useful parameters for the differential diagnosis of disease flares and infections in pediatric-onset SLE patients and to develop predictive calculators that might assist in decision-making in daily clinical practice.

## Results

### Patient and clinical characteristics

Fifty patients who accounted for a total of 185 clinical visits were included in the study (Table 1). Among these 50 patients, 7 (14%) were male and 43 (86%) female; the mean age at enrollment was 13.9 ± 4.4 years old. The type of infections and positive culture results were recorded in Table 2. The most common fungal infections of our study include *Candida* species and *Pneumocystis jirovecii*^25^. These 185 visits were divided into 4 groups: infected-active visits as group A (n = 102; 55%), infected-inactive visits as group B (n = 11; 6%), noninfected-active visits as group C (n = 59; 32%), and noninfected-inactive visits as group D (n = 13; 7%) (Table 3). Categorization of outcomes was performed in a fashion similar to that in previous studies^18,26^. The trend of our CRP results resembled those reported by others^18,26,27^, as did the trends of our ESR and PCT results^18,27^ (Supplement Fig. S1). The infected-active group (group A) had the highest PLR values among all four groups^26^. Without infection, CRP levels are higher in active SLE than in inactive SLE^18,28^.

**Table1:** Characteristics, clinical manifestations, infections patterns, and medications of 50 pediatric SLE patients.

Patients	n = 50
Age (years) at enrollment	13.9 ± 4.4
Male:Female, n (%)	7:43 (14:86)
**Organ involvement during flare visits, n (%)**	
Neuropsychiatric	11 (22)
Vasculitis	9 (18)
Arthritis	24 (48)
Myositis	2 (4)
Renal	36 (72)
Mucocutaneous	36 (72)
Serositis	15 (30)
Hematologic	21 (42)
**Infections, n (%)**	
Respiratory	28 (56)
Skin and soft tissue	4 (8)
Infective endocarditis	1 (2)
Brain abscess	1 (2)
Intra-abdominal infection	5 (10)
Urinary tract infection	10 (20)
Bacteremia	5 (10)
Herpes zoster	3 (6)
**Previous medications, n (%)**	
Hydroxychloroquine	35 (70)
Oral glucocorticoid	38 (76)
Immunosuppressant	34 (68)
Cyclosporine	12 (24)
Azathioprine	8 (16)
Mycophenolic	18 (36)
**Medications during admission, n (%)**	
Steroid pulse therapy	17 (34)
Steroid mini-pulse therapy	17 (34)
Cyclophosphamide pulse therapy	9 (18)
Rituximab	4 (8)

**Table 2:** The affected sites and pathogenic microorganisms in 50 pediatric SLE patients.

Pathogen/affected site	Cultured pathogen species (n)	n (%)
**Bacteria**		**20 (40)**
Pneumonia	Acinetobacter baumannii (2) Pseudomonua aeruginosa (2) Stenotrophomonas maltophilia (1) Ralstonia pickettii (1) Mycoplasma pneumonia (1)	7 (14)
Urinary tract infection	Klebsiella pneumonia (1) Streptococcus agalactiae (1) Pseudomonas aeruginosa (1) Staphylococcus aureus (1) Enterococcus faecium (2) Stenotrophomonas maltophilia (1)	7 (14)
Gastrointestinal infection	Goup B Salmonella (1)	1 (2)
Bacteremia	Streptococcus pneumonia (1) Staphylococcus aureus (1) Stenotrophomonas maltophilia (1) Abiotrophia defective (1) Pseudomonas aeruginosa (1)	5 (10)
**Virus**		**4 (8)**
Herpes Zoster	Herpes Zoster (2)	2 (4)
Pneumonia	Influenza virus A (1) Cytomegalovirus (1)	2 (4)
**Fungus**		**18 (36)**
Oral candidiasis	Candida albicans (4) Candida tropicalis (1) Candida lusitaniae (1)	6 (12)
Pneumonia	Candida tropicalis (1) Candida glabrata (1) Candida albican (2) Pneumocystis jirovecii (1)	5 (10)
Urinary Tract Infection	Candida albicans (4) Yeast like organism (1) Candida tropicalis (1)	6 (12)
Fungemia	Candida tropicalis (1)	1 (2)
**Bacteria + Fungus**		**6 (12)**
Pneumonia	Candida glabrata + Acinetobacter baumannii (1)	1 (2)
	Candida tropicalis + Pseudomonas aeruginosa (1)	1 (2)
	Candida albicans + Stenotrophomonas maltophilia (1)	1 (2)
Urinary Tract Infection	Candida albicans + Enterococcus faecium (1)	1 (2)
	Candida tropicalis + Enterococcus faecium (1)	1 (2)
	Yeast like organism + Pseudomonua aeruginosa (1)	1 (2)

**Table 3:** Comparison 185 clinical visits related to activity flare and/or acute infection.

Group (n = 185)	Infected-active (n = 102)	Infected-inactive (n = 11)	Noninfected-active (n = 59)	Noninfected-inactive (n = 13)
**Laboratory parameters (mean ± SD)**				
WBC (/uL)	8430 ± 5807	6702 ± 3717	8441 ± 5793	10,715 ± 5313
Hb (g/dL)	9.2 ± 1.9	11.4 ± 2.3	10.7 ± 2.0	11.9 ± 1.9
PLT (× 10^3^/uL)	176.8 ± 114.6	224.5 ± 86.0	229.5 ± 135.8	310.6 ± 100.6
Segment (%)	77.7 ± 17.8	77.6 ± 9.5	72.8 ± 14.3	81.2 ± 7.5
Lymphocyte (%)	12.9 ± 11.6	16.3 ± 8.4	19.9 ± 12.7	12.3 ± 5.2
CRP (mg/dL)	2.25 ± 4.08	2.32 ± 2.27	1.02 ± 2.33	0.31 ± 0.42
ESR (mm/h)	41.0 ± 38.4	38.8 ± 18.6	33.5 ± 32.3	29.7 ± 19.5
ESR/CRP	168.7 ± 433.6	94.2 ± 178.5	254.5 ± 387.0	271.5 ± 366.0
Procalcitonin (ng/mL)	1.998 ± 6.232	0.54 ± 1.18	0.27 ± 0.44	0.06 ± 0.04
ANA titer (1:N)	1,303 ± 940	352 ± 476	1,187 ± 966	467 ± 578
C3 (mg/dL)	62.71 ± 31.45	78.18 ± 30.50	58.96 ± 37.94	103.41 ± 24.75
C4 (mg/dL)	13.67 ± 10.09	18.90 ± 10.85	11.23 ± 9.37	13.18 ± 6.17
Anti-dsDNA (IU/mL)	545.18 ± 492.99	601.38 ± 330.58	770.89 ± 418.78	291.57 ± 310.66
NLR	22.19 ± 37.09	8.07 ± 7.66	9.47 ± 19.40	8.73 ± 5.40
PLR	421.0 ± 396.5	302.0 ± 128.8	253.1 ± 177.2	305.0 ± 98.1
RPR	0.15 ± 0.14	0.07 ± 0.02	0.11 ± 0.13	0.06 ± 0.03
**Clinical scoring indices**				
SLEDAI-2K (mean ± SD)	19.68 ± 8.30	9.64 ± 5.53	15.53 ± 8.29	10.15 ± 5.29
Mild activity (< = 6), (n, %)	4 (7%)	4 (36%)	9 (15%)	5 (38%)
Moderate activity (7–12), (n, %)	17 (19%)	3 (28%)	17 (29%)	4 (31%)
Severe activity (> 12), (n, %)	76 (74%)	4 (36%)	33 (56%)	4 (31%)
Renal column (mean)	9.81	3.64	5.69	7.69
CNS column (mean)	2.78	1.09	0.68	0
Vasculitis column (mean)	0.86	0	1.49	0.31
Arthritis column (mean)	0.71	0.36	1.42	0.31
Myositis column (mean)	0.24	0	0	0
Cutaneous column (mean)	0.85	1.27	1.83	0.62
Serositis column (mean)	1.02	0	0.51	0
Complement column (mean)	1.62	0.73	1.66	0.77
SLICC/ACR Damage Index (SDI) (mean, range)	3.01 ( 0–7)	0.64 ( 0–2)	0.69 ( 0–5)	0.92 ( 0–3)

### Parameters predictive of activity flare

**Figure 1:**
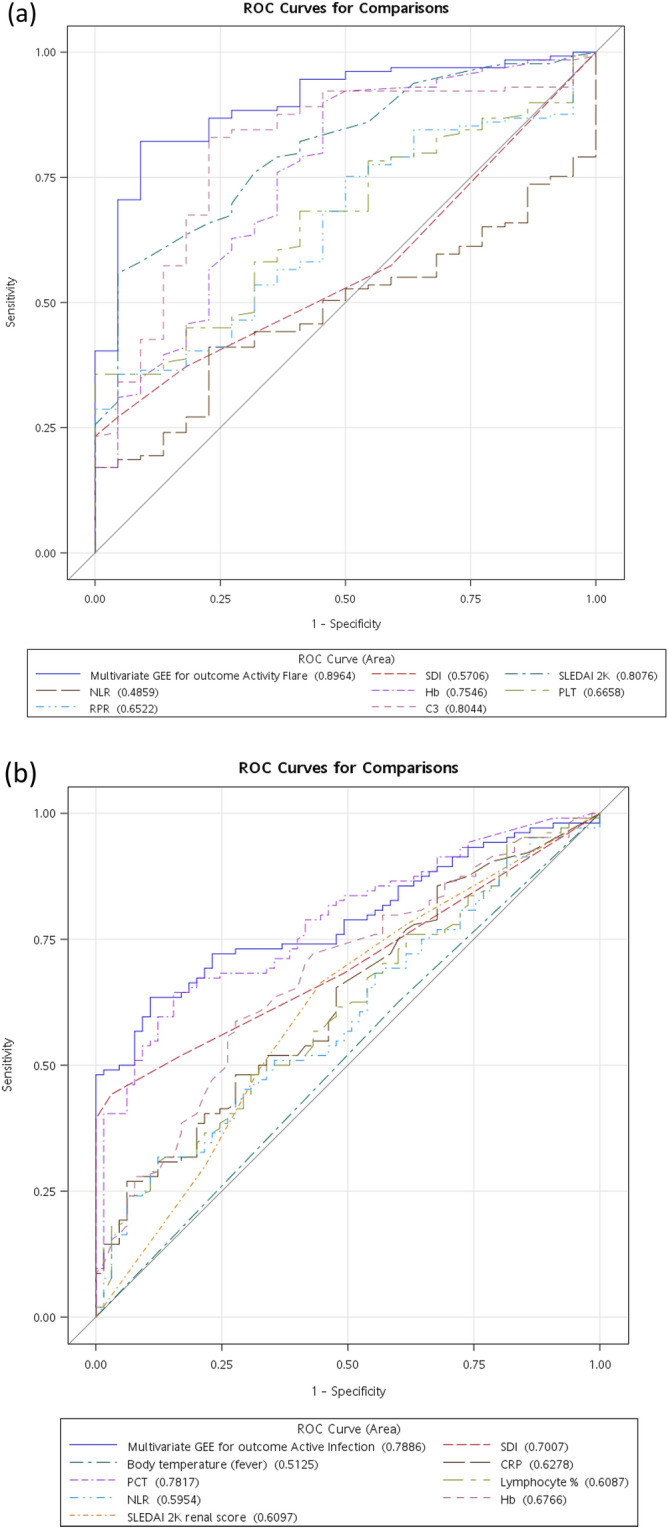
Receiver Operating Characteristic (ROC) curves for prediction of (**a**) activity flares and (**b**) acute infections. In (**a**), ROC for SDI score, SLEDAI 2K score, NLR, Hb levels, platelet levels, RPR, and C3 levels according to univariate GEE result (dashed line), and their combination (multi flare GEE) according to multivariate GEE result (solid line) to predict activity flare is shown. The area under curve (AUC) were shown within parentheses. In (**b**), ROC for SDI score, fever temperature, CRP levels, PCT levels, lymphocyte percentage, NLR, Hb levels, and SLEDAI 2 K renal score according to univariate GEE result (dashed line), and their combination (multi infection GEE) according to multivariate GEE result (solid line) to predict acute infection is shown. The area under curve (AUC) were shown within parentheses.

Among all the parameters analyzed, we found SDI score, SLEDAI 2K score, NLR, RDW-to-platelet ratio (RPR), ANA level, anti-dsDNA level, antiphospholipid Ab level, and urine cast to be positive predictors of activity flare. Conversely, Hb levels (g/dL), platelet levels, C3 levels (mg/dL), C4 levels (mg/dL), and urine nitrate were negatively associated with the occurrence of disease activity (Supplementary Table S1a). Multivariate GEE (generalized estimating equation) analysis showed that SDI score, SLEDAI 2K score, NLR, Hb level (g/dL), platelet level, RPR, and C3 level (mg/dL) were independent parameters for predicting SLE activity flares (Table 4). In this study, we confirmed that the NLR, PLR, and RPR are useful markers for the assessment of disease activity in pediatric SLE patients^23,29^, and the combination of these seven parameters resulted in a model with a calculated AUC of 0.8964 and a sensitivity of 82.2% and specificity of 90.9% (Fig. 1a).

**Table 4:** Multivariate GEE for outcome of activity flare.

Parameter	Estimate	Standard error	95% Confidence limits		*P*-value
SDI	− 0.0148	0.0088	− 0.0320	0.0025	0.0936
SLEDAI 2 K	0.0108	0.0029	0.0050	0.0165	0.0002
NLR	0.0013	0.0004	0.0005	0.0021	0.0010
Hb	− 0.0305	0.0126	− 0.0552	− 0.0057	0.0159
PLT*	− 0.0147	0.0265	− 0.0667	0.0373	0.5797
RPR	0.1614	0.2026	− 0.2357	0.5585	0.4257
C3	− 0.0025	0.0013	− 0.0050	− 0.0001	0.0449

Seven significant effectors are shown.

*NLR* neutrophil-to-lymphocyte ratio; *RPR* RDW-to-platelet ratio.

*Original values divided by 100.

We thus propose an Activity Predict Score formula:

$${\text{Activity}}\;{\text{Predict}}\;{\text{Score}} = {1}.{17}0{7} - 0.0{146} \times {\text{SDI}}\;{\text{score}} + 0.0{1}0{8} \times {\text{SLEDAI}}\;{\text{2K}}\;{\text{score}} + 0.00{13} \times {\text{NLR}} - 0.0{3}0{5} \times {\text{Hb}} - 0.0{147} \times {\text{PLT }}\left( {{\text{original}}\,{\text{value}}\;{\text{divided}}\,{\text{by}}\;{1}00} \right) + 0.{1614} \times {\text{RPR}} - 0.00{25} \times {\text{C3}}.$$

We obtained the largest Youden Index when the cutoff point of the Activity Predict Score was 0.76652; that is, an Activity Predict Score greater than 0.76652 indicates an activity flare, whereas as score less than 0.76652 indicates no activity flare.

$$\text{cut-off point}=\frac{{e}^{-6.3278+10.5225Predict Score}}{1+{e}^{-6.3278+10.5225Predict Score}}$$

### Parameters predictive of acute infection

Using GEE, we found that acute infection was associated with SDI score, fever temperature (°C), CRP level (mg/dL), PCT level (ng/mL), NLR, PLR, and renal score of SLEDAI 2 K but that lymphocyte percentage, Hb level (g/dL), and urine nitrate were negative predictors of infectious events (Supplementary Table S1b). Multivariate GEE analysis showed that SDI score, fever temperature (°C), CRP level (mg/dL), PCT level (ng/mL), lymphocyte percentage, NLR, Hb level (g/dL), and SLEDAI 2 K renal score were independent parameters for predicting acute infection in SLE patients (Table 5). Renal disease, despite being associated with infections in the univariate analysis, did not retain statistical significance in the multivariate analysis in some series^30,31^. However, our result resembled a previous report that renal involvement is significantly associated with active infection, as based on multivariate GEE analysis^32^. Of note, our data were consistent with a previous report that any increase in the SDI was associated with the occurrence of serious infection^31^. We also showed that compared to PCT, CRP is a more sensitive and specific marker for diagnosing bacterial infection in SLE^33^. Regardless, some reports have shown that PCT is more specific and has better diagnostic accuracy than CRP for infection in SLE^15,34,35^. The combination of these eight parameters resulted in a model with a calculated AUC of 0.7886 and a sensitivity of 63.5% and specificity of 89.2% (Fig. 1b).

**Table 5:** Multivariate GEE for outcome of acute infection.

Parameter	Estimate	Standard error	95% Confidence limits		*P*-value
SDI	0.0782	0.0169	0.0451	0.1114	< .0001
Fever temperature	0.0997	0.0947	− 0.0859	0.2853	0.2926
CRP	0.0341	0.0075	0.0195	0.0487	< .0001
PCT	0.0005	0.002	− 0.0035	0.0045	0.8069
Lymphocyte percentage	− 0.0006	0.0034	− 0.0073	0.0061	0.8520
NLR	0.0005	0.0007	− 0.0009	0.0019	0.4840
Hb	− 0.0125	0.0243	− 0.0601	0.0352	0.6086
SLEDAI 2K renal score	0.0086	0.0079	-0.0069	0.0242	0.2769

Eight significant effectors are shown.

*NLR* neutrophil-to-lymphocyte ratio; *PCT* procalcitonin.

Predicted by multiple GEE results, we also obtained the Infection Predict Score:

$${\text{Infection Predict Score}} = 0.4193 + 0.0782 \times {\text{SDI score}} + 0.0997\times\;{\text{fever temperature}} + 0.0341 \times {\text{CRP}} + 0.0005 \times {\text{PCT}} - 0.0006 \times {\text{lymphocyte percentage}} + 0.0005 \times {\text{NLR}} - 0.0125 \times {\text{Hb}} + 0.0086 \times {\text{SLEDAI 2K renal score}}$$

There will be the largest Youden Index will occur at cutoff value of 0.58286; that is, when the Infection Predict Score is greater than 0.58286, acute infection will be classified, whereas no acute infection will be classified at a score less than 0.58286.

$$\text{cut-off point}=\frac{{e}^{-2.9738+6.2104Predict Score}}{1+{e}^{-2.9738+6.2104Predict Score}}$$

### Development of a calculator model to simultaneously differentiate flares from infections

Multinomial logistic regression, which describes the probability of being in a specific group, was used to analyze the individual effects of covariates (independent variables) on discrete nominal outcomes^36^. We selected a total of 10 variables (SDI, SLEDAI 2K, fever temperature, PCT, lymphocyte percentage, NLR, Hb, PLT, RPR, C3) to establish multinomial logistic regression. The regression formula obtained by multinomial logistic regression is as follows:

1. $${\text{ln}}\left( {\uppi {\text{A}}/\uppi {\text{D}}} \right) = 11.932 - 1.3896 \times {\text{SDI score}} + 0.4166 \times {\text{SLEDAI 2K score}} + 9.9529 \times {\text{fever}}\;{\text{temperature}} + 36.4342 \times {\text{PCT}} + 1.028 \times {\text{lymphocyte}}\;{\text{percentage}} + 1.3915 \times {\text{NLR}} - 1.0061 \times {\text{Hb}} - 0.00784 \times {\text{PLT}} - 21.2367 \times {\text{RPR}} - 0.0564 \times {\text{C}}3$$
2. $${\text{ln}}\left( {\uppi {\text{B}}/\uppi {\text{D}}} \right) = 6.1895-1.5815 \times {\text{SDI score}}+0.2968 \times {\text{SLEDAI 2K score}} + 10.9273 \times {\text{fever}}\;{\text{temperature}} + 36.1573 \times {\text{PCT}} + 0.9752 \times {\text{lymphocyte}}\;{\text{percentage}} + 1.3517 \times {\text{NLR}} - 0.9872 \times {\text{Hb}} - 0.0229 \times {\text{PLT}} - 56.2559 \times {\text{RPR}} - 0.0434 \times {\text{C}}3$$
3. $${\text{ln}}\left( {\uppi {\text{C}}/\uppi {\text{D}}} \right) = 12.5014 - 1.7698 \times {\text{SDI score}} + 0.3917 \times {\text{SLEDAI 2K score}} + 10.0168 \times {\text{fever}}\;{\text{temperature}} + 35.6598 \times {\text{PCT}} + 1.0357 \times {\text{lymphocyte}}\;{\text{percentage}} + 1.3925 \times {\text{NLR}} - 0.8858 \times {\text{Hb}} - 0.00679 \times {\text{PLT}} - 18.9057 \times {\text{RPR}} - 0.0646 \times {\text{C}}3$$

By inputting the value of the selected parameters into these three equations, we can obtain the ratio values of π_A_, π_B_, π_C_ and π_D_. If the value obtained is larger, the probability of being classified into that nominal group is greater (the group divided into D is the reference group), and we will classify particular visits into that group (groups A, B, C). That is, if the calculated ln(π_A_/π_D_) is greater than 1 and is the largest number compared with others (ln(π_B_/π_D_) and ln(π_C_/π_D_)), the cases is categorized as in group A. If all three calculated numbers [ln(π_A_/π_D_), ln(π_B_/π_D_), and In(π_C_/π_D_)] are below 1, the case is categorized as in group D. With a combination of these ten parameters, we can simultaneously predict four groups with an accuracy of 70.13% for the infected-active group, 10% for the infected-inactive group, 59.57% for the noninfected-active group, and 84.62% for the noninfected-inactive group. From our multinomial logistic regression analysis, we identified SDI, SLEDAI 2K, fever temperature (°C), PCT, lymphocyte percentage, NLR, Hb (g/dL), PLT (K/μL), RPR, and C3 (mg/dL) as influencing factors for simultaneously differentiating activity flares from acute infections. By knowing the values of the observed parameters, we can predict the group classification of any specific visit for an individual patient.

### Evaluation of possible associated interaction between acute infection and activity flare

**Figure 2:**
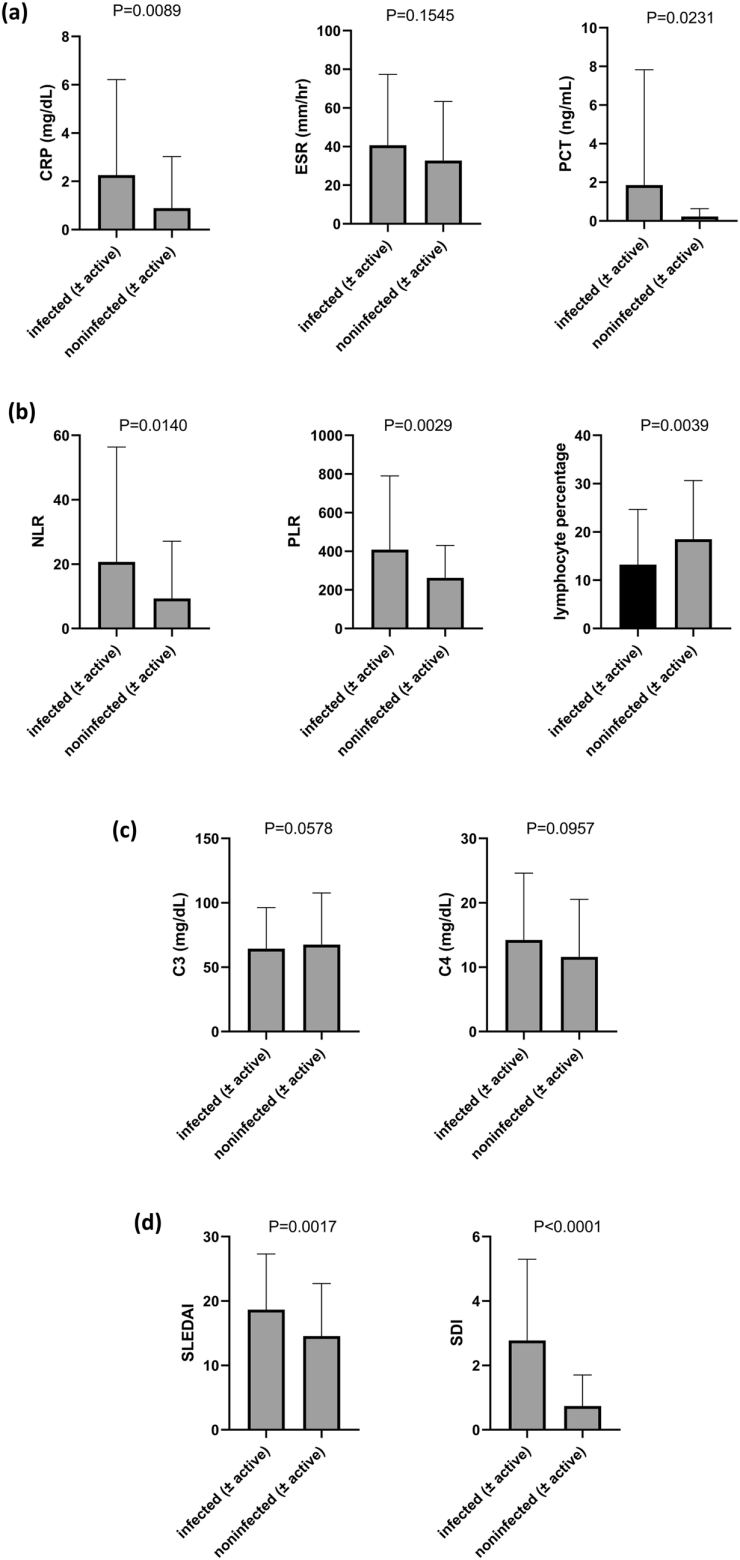
Comparison for mean values of major parameters from infected (infected-active plus infected-inactive; A + B) groups *vs* noninfected (noninfected-active plus noninfected-inactive; C + D) groups. Parameters from four different domains, including (**a**) inflammation (CRP, ESR, PCT), (**b**) hematology (NLR, PLR, lymphocyte percentage), (**c**) complement (C3, C4), and (**d**) clinical status (SLEDAI, SDI), are depicted.

To observe whether there is an associated interaction between acute infection and activity flare, we sought to compare parameters from combined groups with or without infection (Fig. 2). We found that CRP (mg/dL), PCT (ng/mL), lymphocyte percentage, NLR, PLR, SLEDAI 2K and SDI from the combined groups with infection were significantly higher than those of the combined groups without infection. However, ESR (mm/h), C3 (mg/dL) and C4 (mg/dL) were not significantly different between the two combined groups. Our results (elevated SLEDAI 2K, SDI, NLR, and PLR under noninfected conditions) indicate that acute infection might play a triggering role in flare activity^10,37,38^. On the other hand, for proteins that participate in both SLE disease inflammation and acute-phase inflammation, no significant difference in ESR (mm/h), C3 and C4, with or without infection, was observed.

### Trend analysis of parameter changes over time through hospitalization

**Figure 3:**
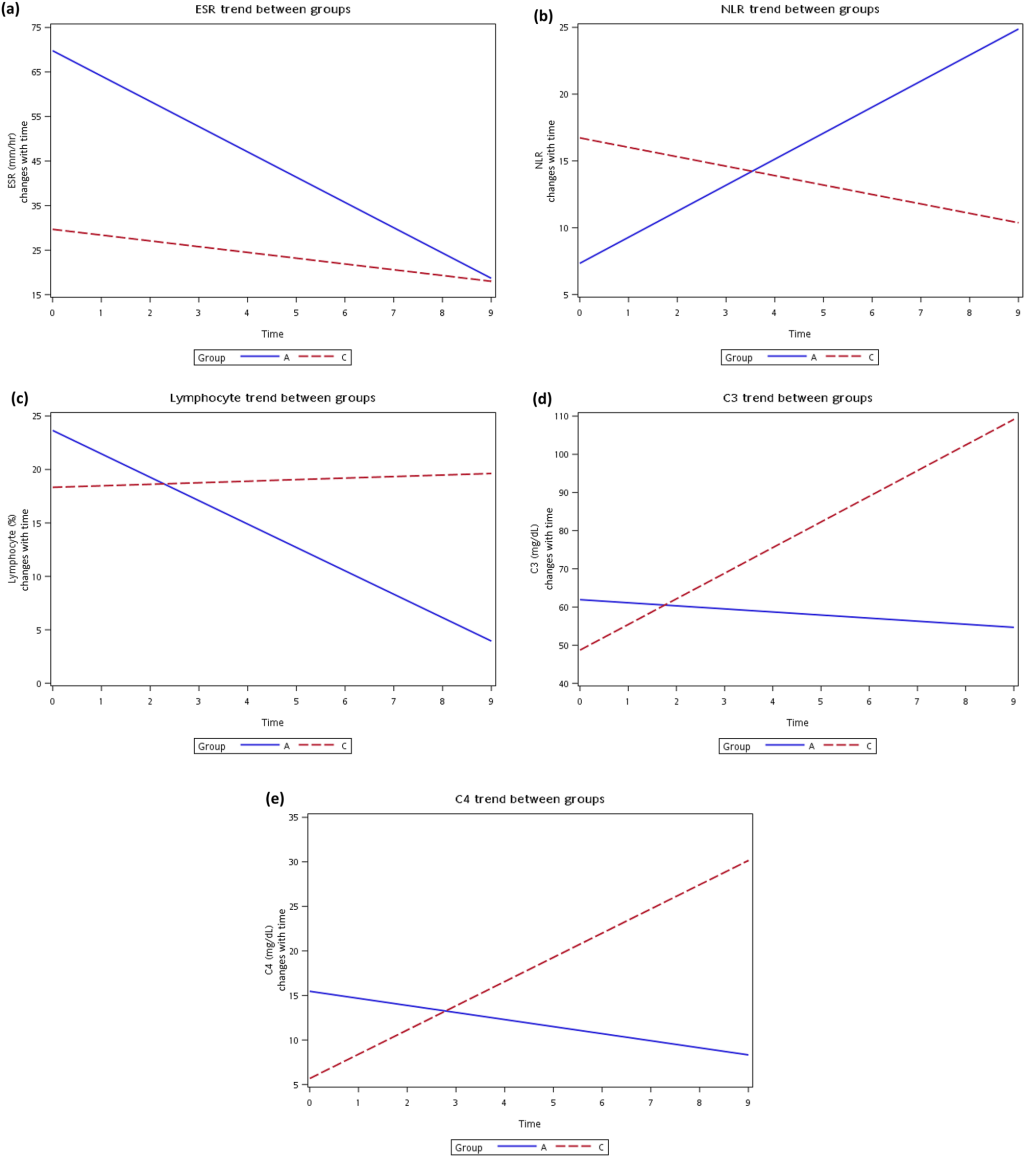
The average changing levels with time of (**a**) ESR, (**b**) NLR, (**c**) lymphocyte percentage, (**d**) C3, and (**e**) C4 during hospitalization are shown from a subset of 24 patients accounting for 29 times of admission. The trend for NLR showed that the NLR in group C were significantly decreased with time while those of group A were significantly increased with time. For lymphocyte percentage trend, the trend in group C was gradually increased with time while those of group A was sharply decreased with time. From (**d**,**e**), we found that the C3 and C4 levels in group C were significantly increased with time while those of group A were significantly decreased with time.

The results of the mean baseline level and changes per time interval of the different groups are shown in Supplement Table S2. There were significant differences in ESR (mm/h), NLR, lymphocyte percentage, C3 (mg/dL), and C4 (mg/dL), as shown in Fig. 3. According to Fig. 3 (a), ESR (mm/h) decreased with time, but the decreasing trend was more prominent in group A than in group C. ESR (mm/h) appears to be a useful biomarker for SLE activity assessment. Indeed, an elevated ESR (mm/h) is included in three of five validated SLE activity scores^28^. Our trend analysis indicated that a higher initial ESR level (mm/h) might reflect the effect of both activity and infection in group A^14^. From the trend difference between groups A and C, we could differentiate noninfected-active SLE visits (group C) from infected-active SLE visits (group A) by the change patterns of ESR (mm/h), NLR, lymphocyte percentage, C3 (mg/dL), and C4 (mg/dL) over time.

## Discussion

**Figure 4:**
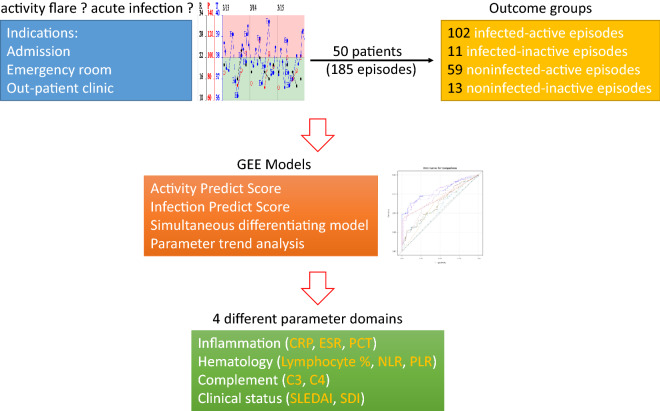
Investigation scheme and brief summary of our study. When SLE patients encounter clinical indications, through the evaluation model based on the parameters of the four main domains, we can make a correct evaluation and appropriate treatment. *CRP* C-reactive protein; *ESR* erythrocyte sedimentation rate; *GEE* generalized estimating equation; *NLR* neutrophil-to-lymphocyte ratio; *PCT* procalcitonin; *PLR* platelet-to-lymphocyte ratio; *SLEDAI* Systemic Lupus Erythematosus Disease Activity Index; *SDI* Systemic Lupus International Collaborating Clinics/American College of Rheumatology Damage Index.

In contrast to previous reports, the most important parameters used to establish the predictive model in our study consisted of four domains under simultaneous evaluation: inflammation (CRP, ESR, PCT), hematology (WBC, PLT, Hb, lymphocyte percentage, NLR, PLR, RPR), the complement system (C3, C4), and clinical status (SLEDAI, SDI) (Fig. 4). This classification approach is conceptually similar to the latest version of the SLE classification criteria^39^. Obtaining several parameters simultaneously remains necessary to differentiate flares from infections^27^. Attribution of clinical manifestations to SLE often requires a comprehensive, multidisciplinary approach to rule out mimics (e.g., infections), taking into account the presence of risk factors (e.g., immunosuppressive therapy), as well as other factors favoring alternative diagnoses (e.g., hematological malignancy)^40^. Cognitive bias might result in diagnostic errors^41^. It seemed that subspecialty expertise does not attenuate this bias. The use of our predictive models may aid in the diagnostic process. We hope to include more molecular biomarkers and genetic signatures in our model in the future, and the related mechanisms will be further explored.

This was a rare study focusing on distinguishing flares from infection in pediatric-onset SLE patients, and the study design closely approximated real-world clinical practice. Studies with designs similar to ours have been reported previously^18,20,21,26,27^. Overall, our study design was similar to those for disease registries derived from the systematic collection of information from patients diagnosed with a particular disease (in this case, SLE). The management of pediatric SLE requires ongoing monitoring of patients, with the collection of data for many parameters/markers. We suggest that this composite predict score can be used in everyday clinical practice to improve the discrimination between activity flare and acute infection in pediatric patients with SLE, who have a greater risk of more fulminant SLE and/or more severe infection, and to take prompt interventions to improve clinical outcomes in pediatric SLE patients.

The NLR and PLR have been used as prognostic indicators for malignancy and are associated with morbidity and mortality in chronic diseases^42^. Previous studies have shown that the NLR is associated with rheumatoid arthritis and psoriasis. Recently, higher NLR and PLR both correlated positively with SLEDAI-2K score and disease activity^23,42,43^. On the other hand, the NLR is associated with infection, and it has been used as an indicator of bacteremia^23,24,26^. Our results showed that the NLR is associated with both disease flare and infection. In adult SLE cohorts, blood cell count ratios appeared to be more informative than blood cell counts per se because pancytopenia and thrombocytopenia tend to occur in SLE^44^. We observed the same tendency in our pediatric-onset SLE patients. Ideally, PLR values should be combined with values for NLR and other inflammatory markers to facilitate a more holistic determination^44^. Here, we demonstrate that the NLR and PLR are important complementary hematological indices that provide additional information about disease activity, the presence of neutrophilic inflammation, infectious complications, disease severity, and organ damage in SLE.

The relationship between infection and SLE disease damage is difficult to evaluate. Infection itself is reported to both potentially facilitate or protect against the development of SLE^11,45^. The results from an adult lupus cohort study in Latin America also showed that increased disease activity and damage accumulation are predictive of infection^31^. One study demonstrated a positive correlation between SDI score and the number of recurrent major infections in pediatric-onset SLE^46^. Sit et al*.* also reported that disease damage was significantly associated with a greater number of episodes of major infection^47^. These findings are consistent with our study of the association between SDI and infection (*P* < 0.0001) using multivariate GEE. Our results also indicate that SDI and SLEDAI-2K renal scores can be used to accurately predict acute infection, consistent with previous reports.

The utility of traditional markers (e.g., CRP, ESR, PCT) for detecting infection in SLE patients has been discussed for several decades. CRP is an acute-phase reactant synthesized by the liver during IL-6 regulation and is known as an inflammatory biomarker^28^. CRP levels reportedly increase during infection, arthritis, and serositis in SLE patients^14^. One study showed that the CRP level is more sensitive and specific in diagnosing bacterial infection than the PCT level^33^. Immune complexes induce severe inflammation via conventional pro-inflammatory pathways, including those for cytokines such as TNF-α and IL-6 (which leads to CRP production). On the other hand, the same immune complexes induce the production of type I IFNs and various immunoregulatory cytokines. The simple consequence is reduced production of CRP in active SLE (group B > group A), despite increased IL-6 levels, which are visible in concomitant infection^18,26–28^. PCT is a precursor peptide of calcitonin associated with invasive bacterial infections. Normally produced by parafollicular C cells, PCT is released in response to bacterial toxins and IL-1β^8^, and PCT has good specificity for distinguishing acute bacterial infection from disease flare in patients with autoimmune diseases, regardless of steroid use. The mechanism underlying PCT production after inflammation and its role are still not completely understood^19^. Furthermore, limited information is available regarding plasma PCT levels in patients with active SLE. Patients with active SLE may have slightly increased PCT levels^15,18^. However, Garvand et al*.* noted an unusual phenomenon, whereby PCT levels were high during macrophage activation syndrome (MAS) episodes in lupus flares^48^, and a systematic literature review suggested that PCT level and SLE disease activity do not correlate^15^. Our comparison of 4 groups showed that the active disease group had higher PCT levels (group A > group B; group C > group D), regardless of the presence or absence of infection, suggesting that activity flares are associated with elevated PCT levels.

In the complement system, C3 and C4 have traditionally been used to assess SLE disease activity. However, studies of C3 and C4 consumption in SLE flares indicate that as markers, C3 and C4 exhibit low sensitivity and a wide range of specificity^12^. One reason for these inconsistent results may be because complement proteins participate in both autoimmune (SLE activity) and inflammation (infection) responses. As a consequence, inflammation due to infection increases, but immune complex consumption during disease activity decreases complement protein levels. Hence, levels of C3 and C4 are regulated by both mechanisms^28^. Interestingly, decreases in C3 or C4 levels are not detected in some patients. Instead, levels can increase relative to the baseline during flare visits^49^. Our results (shown in Fig. 3) indicate that C3 and C4 levels in group C increased significantly over time, which may be related to this phenomenon.

## Conclusions

Infections are a major cause of morbidity and mortality in SLE patients. Infections might mimic and even trigger SLE flares. To distinguish acute infection from activity flare always remains a clinical challenge. The proposed approach (Activity predict score, Infection predict score, and multinomial logistic regression formula) could differentiate flares from infections in pediatric SLE patients. Clinicians could make appropriate judgement and treatment decisions based on the combination of parameters from four different domains simultaneously, including inflammation (CRP, ESR, PCT), hematology (Lymphocyte percentage, NLR, PLR), complement (C3, C4), and clinical status (SLEDAI, SDI) in daily clinical practice (Fig. 4).

## Limitations

This study was preliminary and was limited by that there were only 50 different patients with 185 visits. Further validation, replication and use of the calculator algorithm in larger populations would be indicated and useful to prove its reliability beyond this study. Repeated measures in a single patient may introduces potential bias. More molecular biomarkers and genetic signatures should be included and evaluated in our model to explore related underlying mechanisms. Because autoantibodies are not routine laboratory inspections, so that autoantibody profiles are included in missing data due to insufficient data quantities, which may have some impact on our evaluation results. The differentiation of flares versus infection due to microorganism types and infection sites needs further investigation.

## Methods

### Patients

This was a retrospective study conducted by reviewing the medical records of 50 pediatric-onset (≤ 18-year-old) SLE^50^ patients presenting with 185 clinical visits for any clinical condition from August 1, 2015, to September 1, 2019, at the Department of Pediatrics, National Taiwan University Hospital (NTUH). Patients who did not meet the 1997 ACR criteria for SLE diagnosis and those with overlapping autoimmune diseases or other chronic inflammatory diseases/infections or malignancies were excluded. This study was approved by the Institutional Review Board and Research Ethics Committee of the National Taiwan University Hospital and was conducted in compliance with the protocol for good clinical practices and the principles of the Declaration of Helsinki. Informed consent was obtained from all participants and/or their legal guardians.

Data encompassing both laboratory and clinical parameters, including general laboratory testing for pediatric SLE (indicators of inflammation, autoantibodies, complement, urinalysis, antiphospholipid antibodies)^51^, biomarkers of infection in SLE^19^, SLEDAI, SDI and markers based on previous reports^23,26,29,43,52^, were collected at each visit as a standardized clinical protocol to ensure that the resulting predicting model can be used in everyday clinical practice (detailed items in Supplement Table S1).

### Outcome definitions

Disease flare was defined according to the SELENA-SLEDAI Flare index (SFI)^53^:

- *Mild or moderate flares* were defined as 1 or more of the following: (a) change in SELENA-SLEDAI instrument score of 3 points or more (but not to more than 12); (b) new or worsening discoid, photosensitive, or other rash attributable to lupus (including lupus profundus, cutaneous vasculitis, or bullous lupus), nasopharyngeal ulcers, pleuritis, pericarditis, arthritis, or fever not attributable to infection; c) increase in prednisone but not to > 0.5 mg/kg/day; d) addition of nonsteroidal anti-inflammatory drugs (NSAIDs) or hydroxychloroquine for SLE activity; and (e) ≥ 1.0 increase in physician’s global assessment (PGA) score but not to more than 2.5.
- *Severe flares* were defined as 1 or more of the following: a) change in SELENA-SLEDAI instrument score to greater than 12; b) new or worsening central nervous system involvement, vasculitis, nephritis, myositis, thrombocytopenia (platelet count < 60 × 10^9^ cells/L), or hemolytic anemia (hemoglobin level < 7 g/dL or decrease in hemoglobin level > 3 g/dL over a 2-week period), each requiring doubling of corticosteroid dosage to a final dosage greater than 0.5 mg/kg per day or hospitalization; (c) any SLE manifestation requiring an increase in dosage of prednisone or equivalent drug to greater than 0.5 mg/kg per day, or initiation of therapy with cyclophosphamide, azathioprine, mycophenolate mofetil, or methotrexate; (d) hospitalization for lupus activity; and (e) increase in PGA score to > 2.5.

Acute infection was defined based on the following: (1) microbiological culture/isolation evidence that explained clinical symptoms; (2) clinical symptoms and/or inflammatory syndrome and/or laboratory/serological results that rapidly regressed after starting antimicrobial (antibiotic, antiviral, or antifungal) therapy; (3) confirmation by radiological and/or imaging study; and/or (4) confirmation via an infectious specialist consultation^20,21,27,35^.

### Statistical analysis

The clinical and laboratory characteristics of the four groups were analyzed. Quantitative variables are presented as means ± standard deviations and ranges. Qualitative variables are shown as numbers (*n*) and percentages. The Mann–Whitney test was used instead of the *t*-test for the analysis of nonparametric data. Associations between parameters and the four outcome groups were analyzed using univariate and multivariate generalized estimating equations (GEEs) with an autoregressive (AR) model, as the GEE approach facilitates the analysis of data collected in longitudinal, nested, or repeated-measures designs and produces more efficient and unbiased regression estimates for analyzing measures with non-normal response variables^54^.

For both laboratory and clinical parameters, univariate logistic regressions were then fit to specific outcome models. A multivariate logistic stepwise regression was performed with variables that were significant according to both *P* value and AUC in univariate analysis. Pearson pairwise correlation coefficients were applied to preclude highly correlated parameters in the univariate model^55^. We excluded parameters with data not missing completely at random^56^. Measures for model adequacy were performed using the Akaike information criterion (AIC)^56^. Through univariate analysis and multivariate logistic stepwise regression, the remaining parameters were designated as “significant effectors” of activity flare or acute infection and were used to derive a Predict Score equation. The estimates of individual significant effectors were combined to generate the Predict Score formula. After obtaining the Predict Score equation, we calculated the score for each visit of an individual patient according to the times of repeated measurements. At this time, the "estimated score" is regarded as *x*, and the "activity flare" or “acute infection” is regarded as *y*. We used logistic regression to find the best cutoff point at which the largest Youden Index (that is, the sum of sensitivity plus specificity is the largest) would be obtained.

SLE is characterized by a relapsing–remitting course between at least two discrete clinical/laboratory episodes, separated by periods of clinical quiescence^57^. Therefore, we assumed that discrete episodes were all conceptually independent. We selected parameters with significant *P* values in univariate analysis and excluded highly correlated parameters using Pearson pairwise correlation analysis. A total of 10 variables were used for modeling. We thus used a multinomial logistic regression (MLR) model for nominal outcomes by setting group D (noninfected-inactive group) as the reference group and developed three regression equations for simultaneous prediction^36^. The relationship between the other outcome (for group A, B, C) and any particular explanatory variable (clinical and laboratory parameters) was captured using particular parameters that define the log odds of response jumping from the reference outcome (group D) to the otherwise outcome (group A, B, C)^36^.

Twenty-four hospitalized patients presented 29 admissions with serial measurement of parameters during admission. The evaluation at the first time point provided baseline data, and the data collected at each ensuing time point were assigned into one of the following 10 time periods: baseline/day 0 (assigned as time point 0), < 3 days (time point 1), 3–5 days (time point 2), 6–10 days (time point 3), 11–15 days (time point 4), 16–20 days (time point 5), 21–25 days (time point 6), 26–30 days (time point 7), 31–35 days (time point 8), and 36–40 days (time point 9). To identify potential markers, we analyzed group-specific parameter trends, i.e., baseline data and data from subsequent assessments in groups A vs C using a multivariate GEE with the AR model and the GLIMMIX procedure. To track trends in changes in laboratory and clinical parameters in groups A and C over time, we excluded the parameters that had estimates not statistically significant, and we utilized the GLIMMIX procedure using 13 variables (WBC, Hb, PLT, lymphocyte, NLR, PLR, RPR, SLEDAI 2K, C3, C4, CRP, PCT, and ESR), employing verification steps such as the type III tests of fixed effects to provide a solution for fixed effects. The default optimization technique for generalized linear mixed models is the quasi-Newton method. Dimensions included G-side covariance parameters. Fit statistics were calculated using generalized chi-square tests. Covariance parameter estimates and fixed-effect solutions were calculated. The analyses were performed using SAS version 9.1 (SAS Institute Inc., Cary, NC).

## Supplementary information

Supplementary Information.

## Abbreviations

AR: Autoregressive
CRP: C-reactive protein
ESR: Erythrocyte sedimentation rate
GEE: Generalized estimating equation
NLR: Neutrophil-to-lymphocyte ratio
PCT: Procalcitonin
PLR: Platelet-to-lymphocyte ratio
RPR: RDW-to-platelet ratio
SLE: Systemic lupus erythematosus
SLEDAI: Systemic Lupus Erythematosus Disease Activity Index
SDI: Systemic Lupus International Collaborating Clinics/American College of Rheumatology Damage Index
