# Supplementary material for: Differential parameters between activity flare and acute infection in pediatric patients with systemic lupus erythematosus
Source: Sci Rep. 2020 Nov 16;10:19913. doi: 10.1038/s41598-020-76789-6 (PMC7670442; doi:10.1038/s41598-020-76789-6)

**Differential Parameters between Activity Flare and Acute Infection in Pediatric Patients with Systemic Lupus Erythematosus**

Kai-Ling Luo**^1^**, Yao-Hsu Yang**^2^**, Yu-Tsan Lin**^2^**, Ya-Chiao Hu**^2^**, Hsin-Hui Yu**^2^**, Li-Chieh Wang**^2^**, Bor-Luen Chiang**^2,^** **^3^** and Jyh-Hong Lee**^2,^** ***

**^1^** Department of Pediatrics, Cathay General Hospital, Taipei, 10630, Taiwan, Republic of China.

**^2^** Department of Pediatrics, National Taiwan University Hospital and National Taiwan University College of Medicine, Taipei, 10002, Taiwan, Republic of China.

**^3^** Graduate Institute of Clinical Medicine, National Taiwan University College of Medicine, Taipei, 10002, Taiwan, Republic of China.

**Correspondence:*

*Dr. Jyh-Hong Lee, Department of Pediatrics, National Taiwan University Hospital, 8 Chung-Shan South Road, Taipei 10002, Taiwan, Republic of China.*

*E-mail address: leonid@ntu.edu.tw*

*Tel: 886-2-23123456 ext. 71719*

*Fax: 886-2-23119087*

**Supplement Table 1a**: Univariate analysis of the association between parameters and activity flare, as determined by the GEE method.

| Parameter | Estimate | Standard Error | 95%  Confidence Limits | | Z | *P*-value | AUC | Standard Error | 95% Wald  Confidence Limits | |
| --- | --- | --- | --- | --- | --- | --- | --- | --- | --- | --- |
| SDI score | 0.0226 | 0.0082 | 0.0066 | 0.0387 | 2.76 | 0.0057 | 0.6112 | 0.0453 | 0.5225 | 0.7000 |
| SLEDAI 2K score | 0.0125 | 0.0031 | 0.0064 | 0.0187 | 4.00 | <.0001 | 0.7937 | 0.0422 | 0.7111 | 0.8764 |
| fever temperature | 0.0705 | 0.0564 | -0.0400 | 0.1810 | 1.25 | 0.2111 | 0.5116 | 0.0543 | 0.4052 | 0.618 |
| CRP | 0.0066 | 0.0038 | -0.0009 | 0.0140 | 1.72 | 0.0854 | 0.4839 | 0.0568 | 0.3726 | 0.5952 |
| ESR | 0.0004 | 0.0004 | -0.0005 | 0.0012 | 0.81 | 0.4155 | 0.4706 | 0.0536 | 0.3654 | 0.5757 |
| ESR/CRP * | -0.0022 | 0.0058 | -0.0135 | 0.0092 | -0.37 | 0.7094 | 0.5448 | 0.0563 | 0.4345 | 0.6552 |
| PCT | 0.0018 | 0.0013 | -0.0009 | 0.0044 | 1.30 | 0.1942 | 0.7548 | 0.0522 | 0.6525 | 0.8571 |
| WBC * | 0.0001 | 0.0004 | -0.0008 | 0.0009 | 0.13 | 0.8956 | 0.4642 | 0.0599 | 0.3468 | 0.5815 |
| lymphocyte percentage | 0.0003 | 0.0013 | -0.0023 | 0.0030 | 0.26 | 0.7969 | 0.4653 | 0.0510 | 0.3653 | 0.5653 |
| monocyte percentage | 0.0022 | 0.0026 | -0.0029 | 0.0072 | 0.84 | 0.4018 | 0.5576 | 0.0556 | 0.4486 | 0.6667 |
| NLR | 0.0008 | 0.0002 | 0.0003 | 0.0012 | 3.20 | 0.0014 | 0.5158 | 0.0511 | 0.4157 | 0.6159 |
| Hb | -0.0426 | 0.0149 | -0.0718 | -0.0133 | -2.85 | 0.0043 | 0.7704 | 0.0545 | 0.6637 | 0.8772 |
| PLT * | -0.0428 | 0.0201 | -0.0823 | -0.0033 | -2.12 | 0.0336 | 0.7011 | 0.0487 | 0.6057 | 0.7965 |
| PLR * | 0.0048 | 0.0029 | -0.0010 | 0.0105 | 1.63 | 0.1022 | 0.4565 | 0.0487 | 0.3611 | 0.5520 |
| RPR | 0.2794 | 0.1169 | 0.0503 | 0.5085 | 2.39 | 0.0168 | 0.6908 | 0.0516 | 0.5896 | 0.7920 |
| ANA * | 0.0399 | 0.0070 | 0.0263 | 0.0536 | 5.74 | <.0001 | 0.8497 | 0.0705 | 0.7115 | 0.9879 |
| Anti-dsDNA* | 0.0167 | 0.0073 | 0.0023 | 0.0311 | 2.27 | 0.0229 | 0.6092 | 0.0571 | 0.4973 | 0.7210 |
| C3 | -0.0031 | 0.0013 | -0.0056 | -0.0006 | -2.43 | 0.0152 | 0.7969 | 0.0503 | 0.6983 | 0.8956 |
| C4 | -0.0080 | 0.0033 | -0.0144 | -0.0016 | -2.46 | 0.0139 | 0.6274 | 0.0591 | 0.5116 | 0.7432 |
| antiphospholipid Ab | 0.1471 | 0.0565 | 0.0364 | 0.2577 | 2.60 | 0.0092 | 0.6548 | 0.0361 | 0.5840 | 0.7255 |
| SLEDAI 2K (Renal) | 0.0077 | 0.0042 | -0.0005 | 0.0159 | 1.83 | 0.0672 | 0.6210 | 0.0562 | 0.5109 | 0.7311 |
| urine cast | 0.1849 | 0.0703 | 0.0471 | 0.3227 | 2.63 | 0.0085 | 0.6323 | 0.0478 | 0.5386 | 0.7260 |
| proteinuria | 0.0837 | 0.0959 | -0.1043 | 0.2716 | 0.87 | 0.3829 | 0.5485 | 0.0599 | 0.4312 | 0.6658 |
| hematuria | 0.0553 | 0.1016 | -0.1437 | 0.2543 | 0.54 | 0.5861 | 0.5326 | 0.0620 | 0.4111 | 0.6541 |
| pyuria | .01350 | 0.0881 | -0.1593 | 0.1862 | 0.15 | 0.8785 | 0.5256 | 0.0609 | 0.4061 | 0.6450 |
| urine nitrate | -0.3541 | 0.0441 | -0.4404 | -0.2678 | -8.04 | <.0001 | 0.5176 | 0.0246 | 0.4694 | 0.5658 |
| urine leukocyte esterase | -0.0845 | 0.1023 | -0.2850 | 0.1160 | -0.83 | 0.4089 | 0.5141 | 0.0528 | 0.4107 | 0.6176 |
| active infection | 0.0468 | 0.0824 | -0.1146 | 0.2082 | 0.57 | 0.5699 | 0.5974 | 0.0562 | 0.4873 | 0.7075 |

*: original values divided by 100.

NLR: neutrophil-to-lymphocyte ratio; PLR: platelet-to-lymphocyte ratio;

PCT: procalcitonin; PLR: platelet-to-lymphocyte ratio; RPR: RDW-to-platelet ratio.

¶: serum albumin level and steroid dosage were excluded.

**Supplement Table 1b**: Univariate analysis of the association between parameters and acute infection, as determined by the GEE method.

| Parameter | Estimate | Standard Error | 95%  Confidence Limits | | Z | *P*-value | AUC | Standard Error | 95% Wald  Confidence Limits | |
| --- | --- | --- | --- | --- | --- | --- | --- | --- | --- | --- |
| SDI score | 0.0907 | 0.0114 | 0.0683 | 0.1131 | 7.94 | <.0001 | 0.7208 | 0.0348 | 0.6527 | 0.7890 |
| SLEDAI 2K score | 0.0097 | 0.0056 | -0.0012 | 0.0206 | 1.74 | 0.0820 | 0.6082 | 0.0422 | 0.5256 | 0.6909 |
| fever temperature | 0.1701 | 0.0854 | 0.0027 | 0.3375 | 1.99 | 0.0464 | 0.4857 | 0.0371 | 0.4130 | 0.5584 |
| CRP | 0.0339 | 0.0095 | 0.0152 | 0.0525 | 3.56 | 0.0004 | 0.6141 | 0.0421 | 0.5316 | 0.6966 |
| ESR | 0.0029 | 0.0015 | -0.0001 | 0.0059 | 1.92 | 0.0548 | 0.5516 | 0.0468 | 0.4600 | 0.6433 |
| ESR/CRP * | -0.0223 | 0.0147 | -0.0511 | 0.0065 | -1.52 | 0.1296 | 0.6692 | 0.0444 | 0.5821 | 0.7563 |
| PCT | 0.0125 | 0.0037 | 0.0052 | 0.0198 | 3.35 | 0.0008 | 0.7876 | 0.0331 | 0.7227 | 0.8525 |
| WBC * | -0.0002 | 0.0006 | -0.0014 | 0.0010 | -0.29 | 0.7748 | 0.5330 | 0.0441 | 0.4466 | 0.6193 |
| lymphocyte percentage | -0.0065 | 0.0026 | -0.0117 | -0.0013 | -2.45 | 0.0144 | 0.6320 | 0.0416 | 0.5504 | 0.7136 |
| monocyte percentage | -0.0072 | 0.0119 | -0.0306 | 0.0161 | -0.61 | 0.5423 | 0.5098 | 0.0433 | 0.4248 | 0.5947 |
| NLR | 0.0022 | 0.0009 | 0.0005 | 0.0039 | 2.49 | 0.0127 | 0.6128 | 0.0424 | 0.5298 | 0.6959 |
| Hb | -0.0608 | 0.0192 | -0.0985 | -0.0231 | -3.16 | 0.0016 | 0.6966 | 0.0392 | 0.6198 | 0.7734 |
| PLT * | -0.0540 | 0.0356 | -0.1238 | 0.0158 | -1.52 | 0.1293 | 0.6482 | 0.0421 | 0.5656 | 0.7308 |
| PLR * | 0.0234 | 0.0084 | 0.0069 | 0.0399 | 2.78 | 0.0055 | 0.5779 | 0.043 | 0.4936 | 0.6621 |
| RPR | 0.1173 | 0.2555 | -0.3835 | 0.6180 | 0.46 | 0.6462 | 0.6631 | 0.042 | 0.5807 | 0.7455 |
| ANA * | -0.0013 | 0.0076 | -0.0162 | 0.0135 | -0.18 | 0.8590 | 0.5231 | 0.0772 | 0.3719 | 0.6744 |
| Anti-dsDNA * | -0.0159 | 0.0108 | -0.0370 | 0.0052 | -1.48 | 0.1399 | 0.6009 | 0.0498 | 0.5033 | 0.6985 |
| C3 | 0.0001 | 0.0014 | -0.0026 | 0.0027 | 0.04 | 0.9686 | 0.5153 | 0.0489 | 0.4194 | 0.6111 |
| C4 | 0.0058 | 0.0042 | -0.0025 | 0.0142 | 1.37 | 0.1692 | 0.5792 | 0.046 | 0.489 | 0.6693 |
| antiphospholipid Ab | 0.3101 | 0.1679 | -0.0189 | 0.6392 | 1.85 | 0.0647 | 0.5639 | 0.0682 | 0.4302 | 0.6976 |
| SLEDAI 2K (Renal) | 0.0191 | 0.0075 | 0.0043 | 0.0339 | 2.53 | 0.0114 | 0.6217 | 0.0421 | 0.5392 | 0.7041 |
| serum albumin level | 0.0026 | 0.0782 | -0.1506 | 0.1559 | 0.03 | 0.9730 | 0.4203 | 0.0509 | 0.3205 | 0.5201 |
| steroid dosage (mg) * | 0.0080 | 0.0068 | -0.0053 | 0.0212 | 1.18 | 0.2388 | 0.6246 | 0.0411 | 0.5441 | 0.7051 |
| urine cast | 0.0739 | 0.0630 | -0.0495 | 0.1974 | 1.17 | 0.2404 | 0.5058 | 0.0478 | 0.4121 | 0.5995 |
| proteinuria | 0.2141 | 0.1127 | -0.0069 | 0.4350 | 1.90 | 0.0576 | 0.5218 | 0.0461 | 0.4315 | 0.6120 |
| hematuria | 0.1694 | 0.1729 | -0.1694 | 0.5083 | 0.98 | 0.3271 | 0.5499 | 0.0497 | 0.4524 | 0.6473 |
| pyuria | 0.1117 | 0.1000 | -0.0843 | 0.3077 | 1.12 | 0.2639 | 0.5325 | 0.0493 | 0.4360 | 0.6291 |
| urine nitrate | -0.6218 | 0.0475 | -0.7150 | -0.5287 | -13.08 | <.0001 | 0.5204 | 0.0143 | 0.4924 | 0.5484 |
| urine leukocyte esterase | 0.1780 | 0.1063 | -0.0303 | 0.3864 | 1.67 | 0.0940 | 0.5701 | 0.0402 | 0.4914 | 0.6488 |
| activity flare | 0.0602 | 0.1626 | -0.2585 | 0.3790 | 0.37 | 0.7110 | 0.5444 | 0.0263 | 0.4929 | 0.5959 |

*: original values divided by 100.

NLR: neutrophil-to-lymphocyte ratio; PLR: platelet-to-lymphocyte ratio;

PCT: procalcitonin; PLR: platelet-to-lymphocyte ratio; RPR: RDW-to-platelet ratio.

**Supplement Table 2**. Trend of changes of parameters with time between infected-active and noninfected-active group.

|  | baseline (group A) | changes with time  (group A) | *P* | baseline (group C) | changes with time  (group C) | *P* |
| --- | --- | --- | --- | --- | --- | --- |
| CRP | 3.4261  ± 0.9745 | − 0.3520  ± 0.2375 | 0.1503 | 3.0388  ± 1.1355 | − 0.4885  ± 0.2784 | 0.1071 |
| ESR | 69.7856  ± 9.8710 | −5.6793  ± 1.9652**^**^** | 0.0098**^**^** | 29.6694  ± 8.4964 | −1.2972  ± 1.9559 | 0.5209 |
| PCT | 4.2391  ± 2.3964 | −0.4995  ± 0.5936 | 0.4078 | 0.4373  ± 0.2172 | 0.003822  ± 0.05330 | 0.9440 |
| NLR | 7.3342  ± 4.7068 | 1.9490  ± 0.8709**^*^** | 0.0344**^*^** | 16.7241  ± 9.5408 | −0.7061  ± 0.8099 | 0.3991 |
| C3 | 68.3852  ± 9.8909 | −1.4593  ± 1.6117 | 0.3750 | 56.0341  ± 12.0172 | 4.3604  ± 1.6232**^*^** | 0.0228**^*^** |
| C4 | 17.4949  ± 2.9071 | −1.0325  ± 0.5254 | 0.0622 | 8.8012  ± 2.4995 | 1.8141  ± 0.5496**^**^** | 0.0080**^**^** |
| WBC | 7141.29  ± 1449.19 | 413.80  ± 324.24 | 0.2132 | 9974.01  ± 2286.48 | 96.2393  ± 497.71 | 0.8497 |
| Lymphocyte % | 23.6510  ± 3.4295 | −2.1898  ± 0.7961**^*^** | 0.0109**^*^** | 18.3290  ± 4.1006 | 0.1438  ± 0.9246 | 0.8788 |
| Hb | 9.8171  ± 0.5198 | 0.05070  ± 0.09942 | 0.6144 | 10.9898  ± 0.7849 | 0.02654  ± 0.1346 | 0.8467 |
| PLT (K) | 198.07  ± 33.3024 | −0.5239  ± 7.1729 | 0.9423 | 237.62  ± 55.9752 | −10.0881  ± 11.4234 | 0.3932 |
| PLR | 271.20  ± 86.7557 | 22.3365  ± 21.5157 | 0.3091 | 184.77  ± 47.9109 | 1.2046  ± 10.1667 | 0.9075 |
| RPR | 0.1485  ± 0.03100 | −0.00597  ± 0.00711 | 0.4090 | 0.1860  ± 0.07277 | −0.00218  ± 0.00846 | 0.8007 |
| SLEDAI 2K score | 19.7354  ± 2.4521 | −0.1715  ± 0.4203 | 0.6865 | 15.2071  ± 2.8083 | −0.8460  ± 0.5543 | 0.1509 |

**^*^**: *p* < 0.05.

**^**^**: *p* < 0.01.

**Supplement Figure 1.** Comparison of mean values of (a) CRP, (b) ESR, (c) Procalcitonin, (d) NLR, (e) PLR, (f) C3, (g) C4, (h) SDI, and (i) SLEDAI in 4 SLE episode groups.


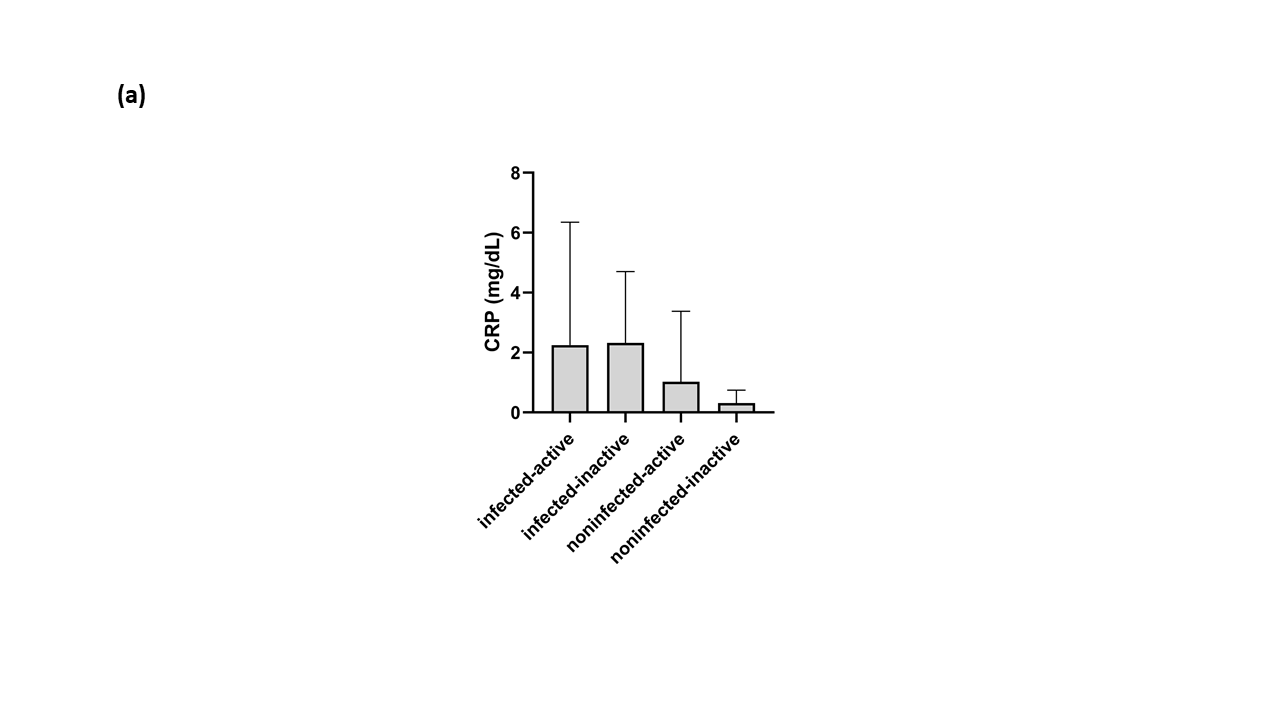


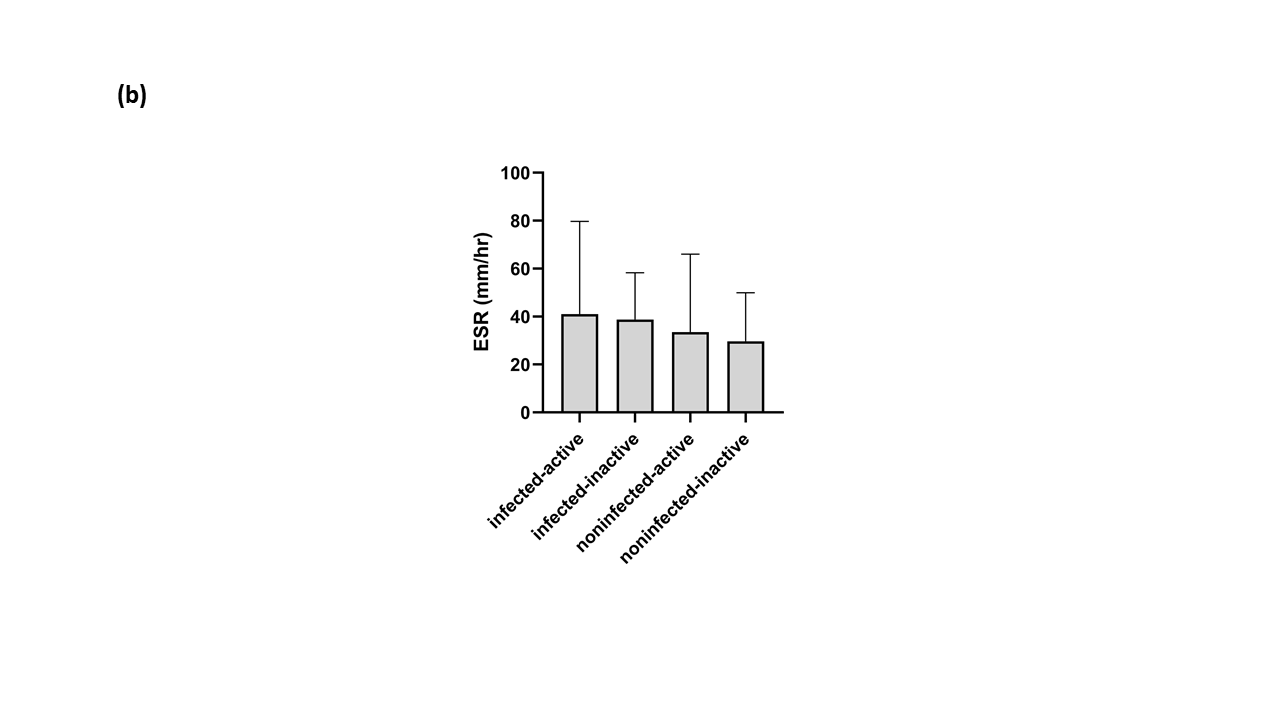


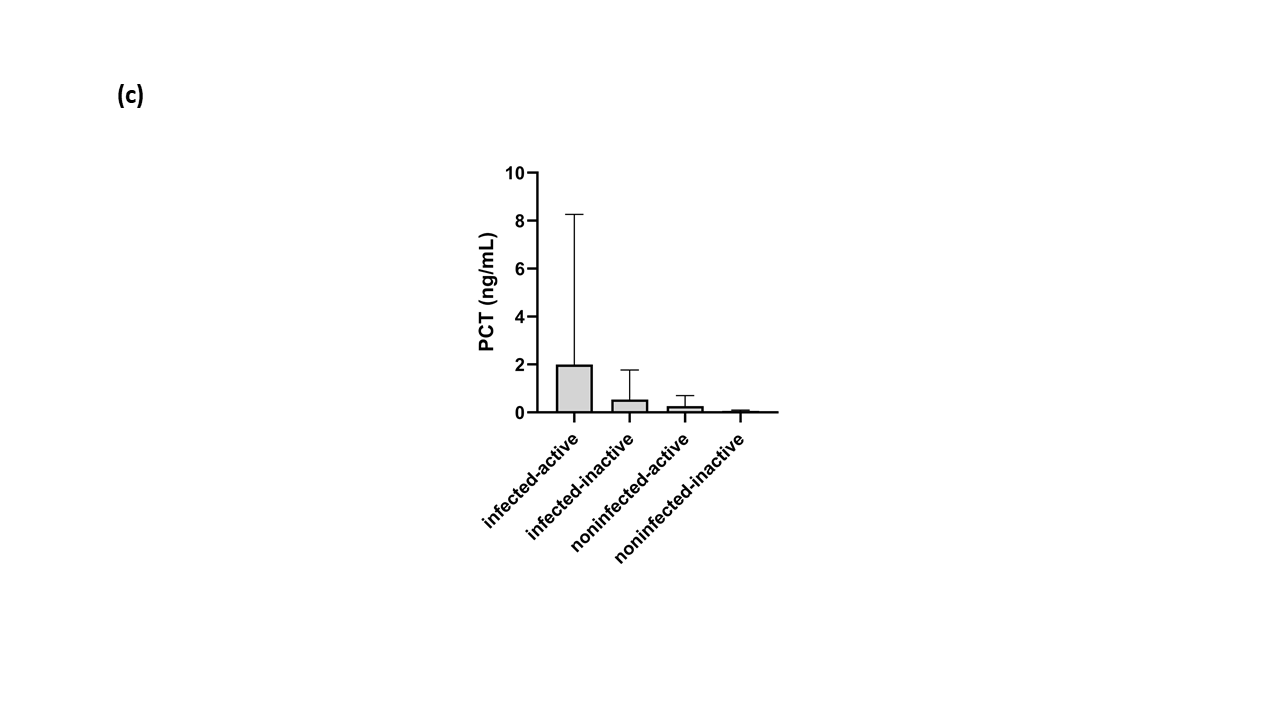


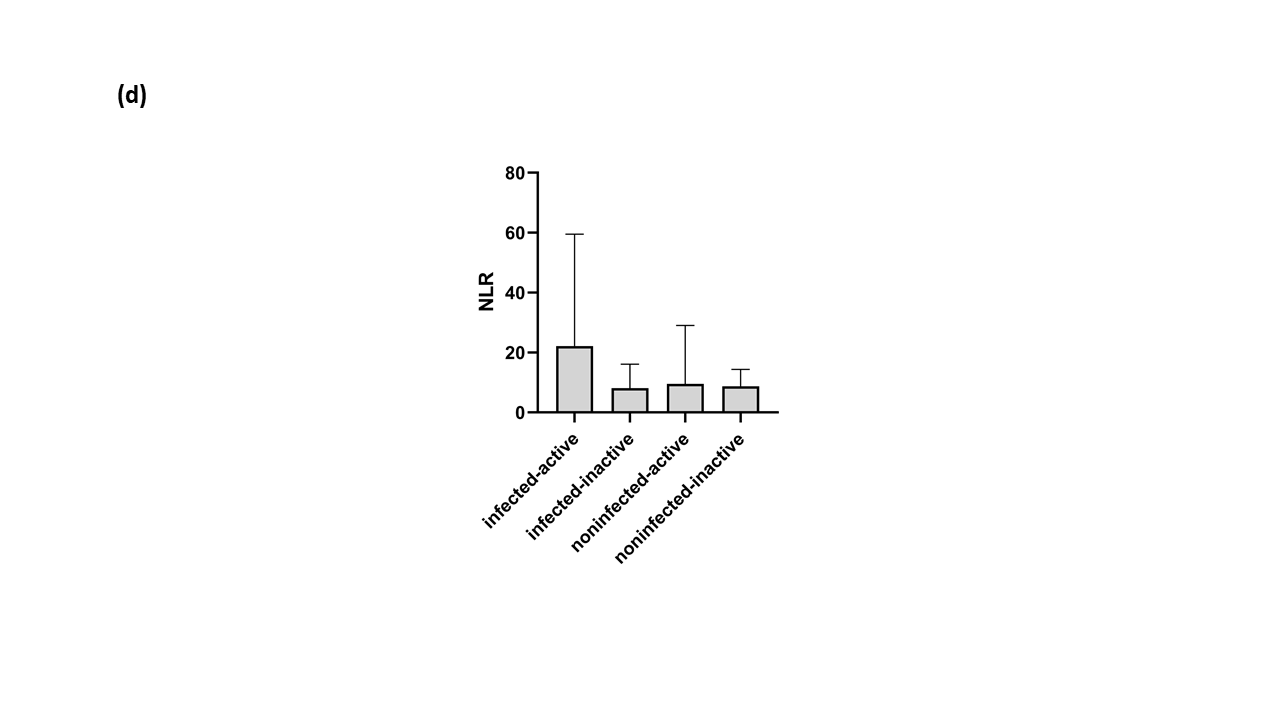


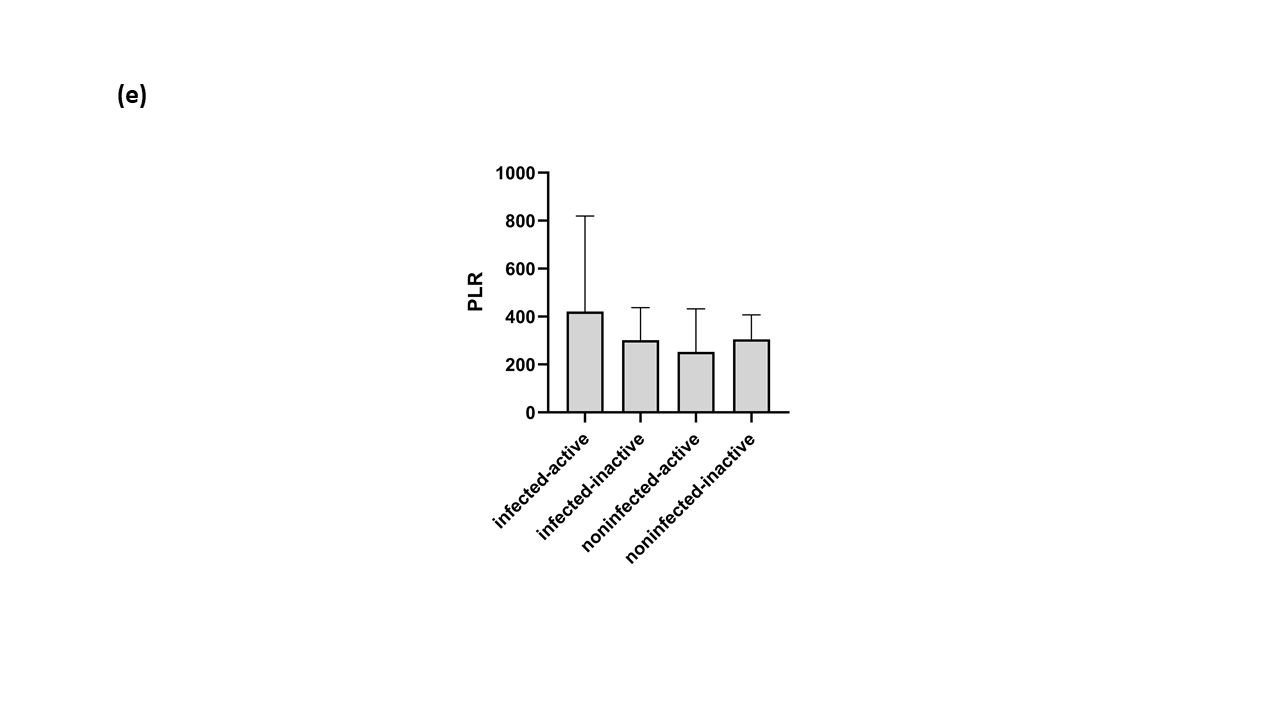


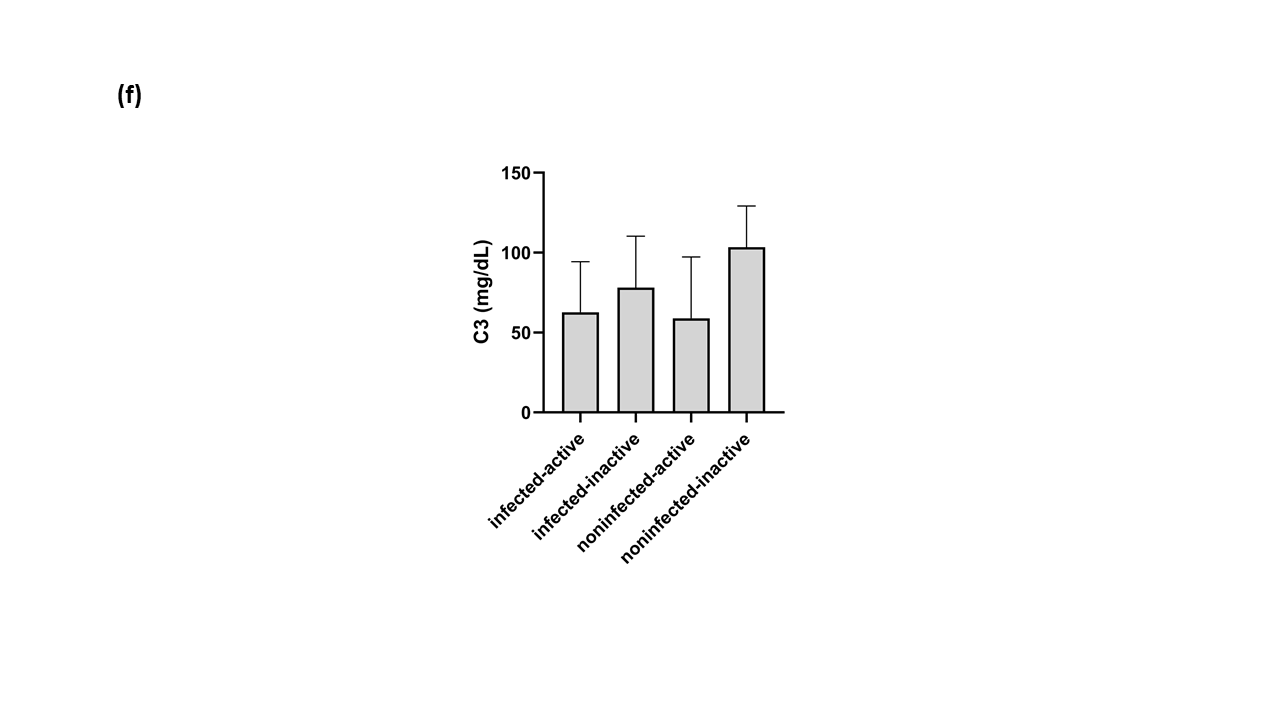


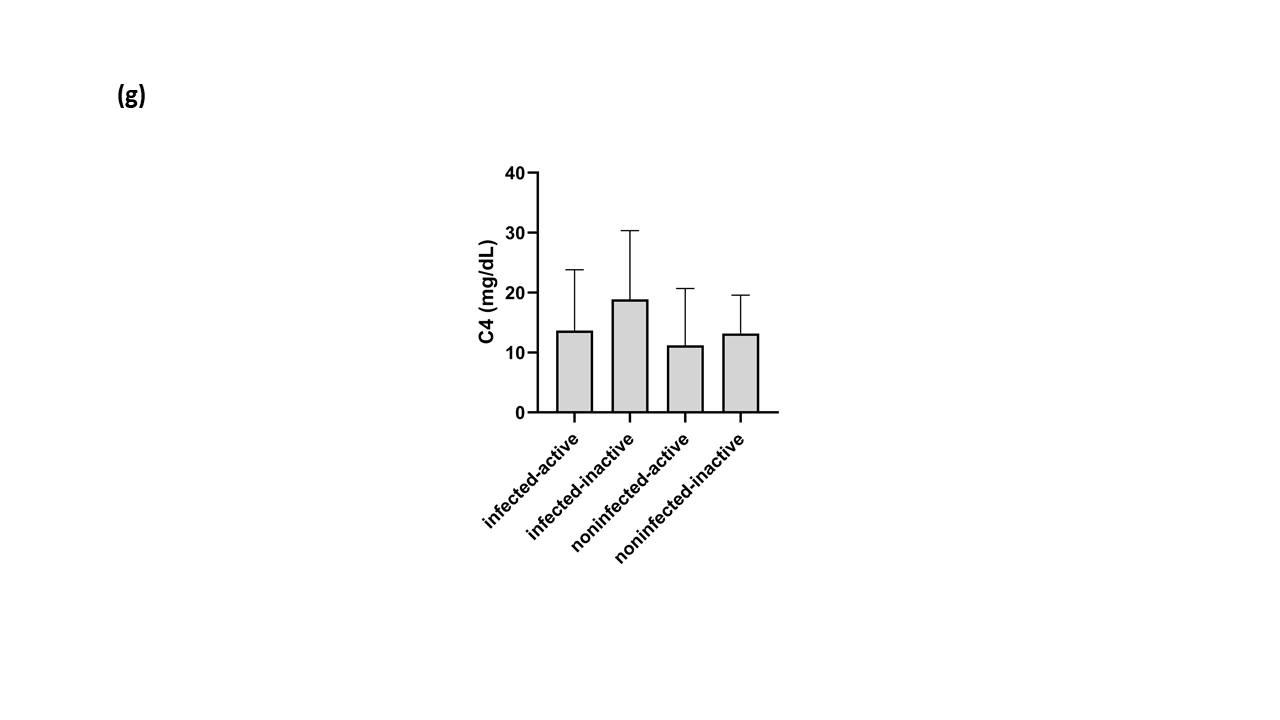


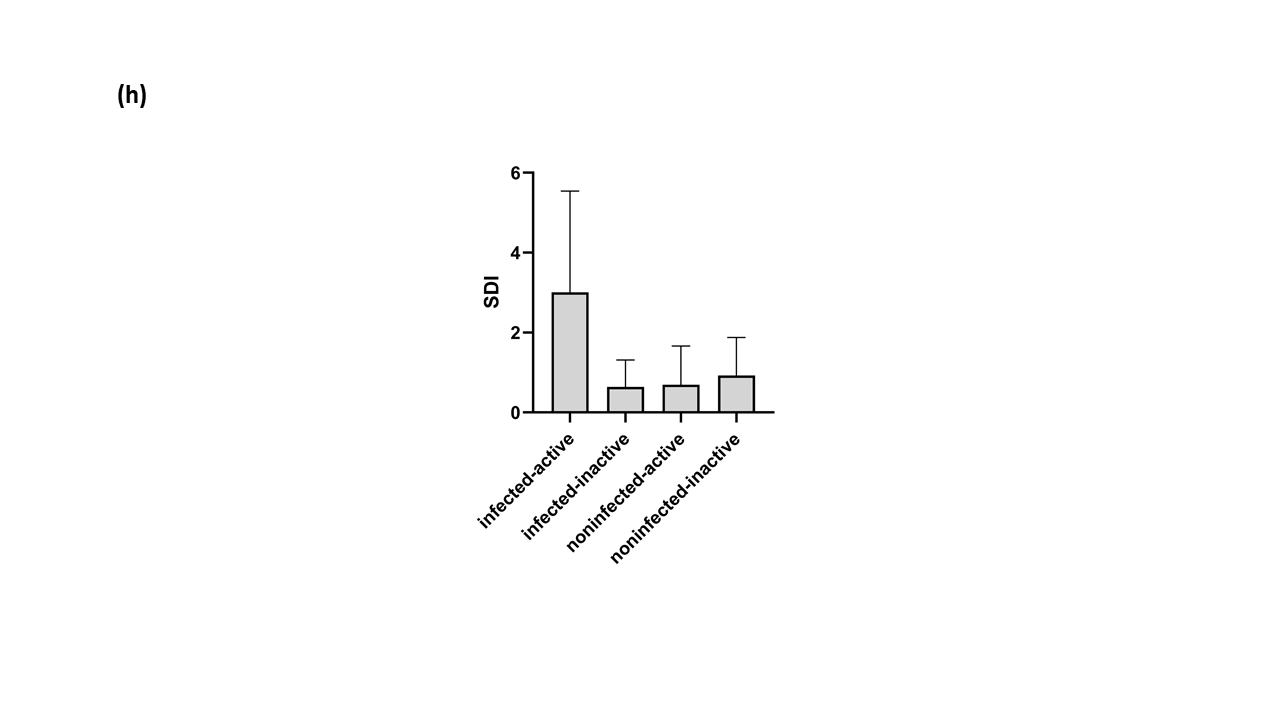


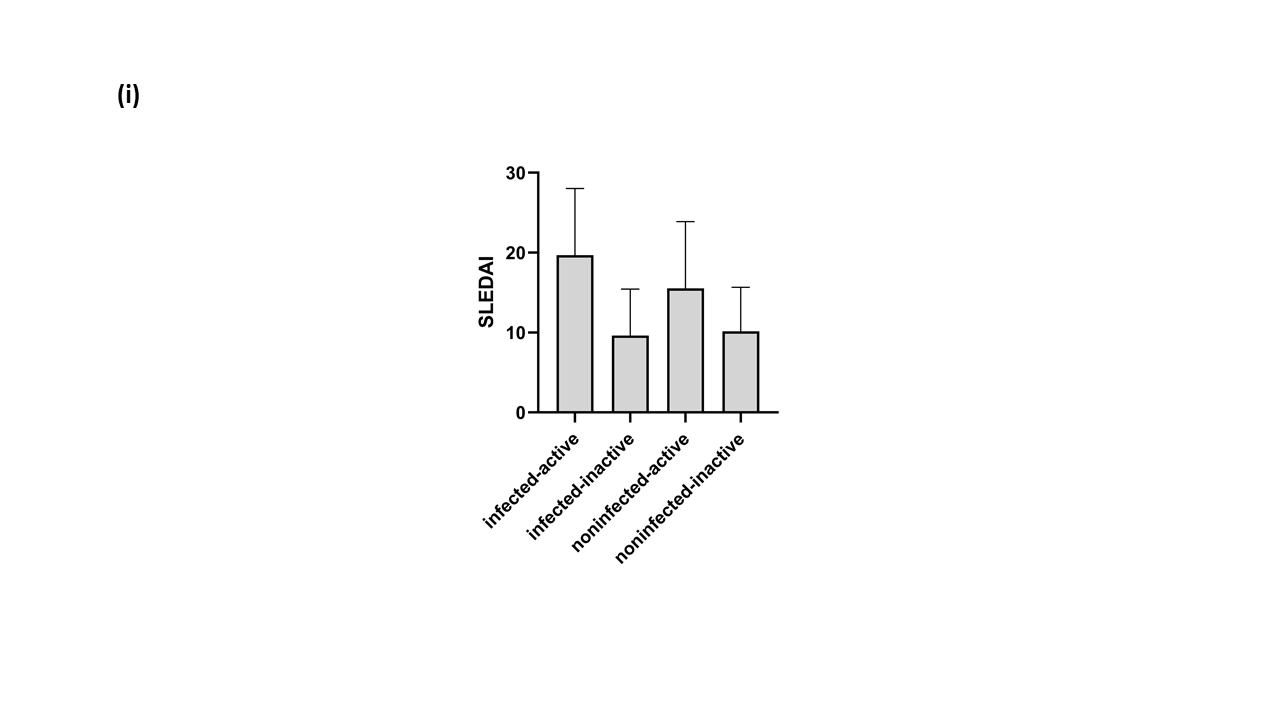

Supplement: Supplementary file 1 — Supplementary Information. [file 41598_2020_76789_MOESM1_ESM.docx]
